# SARS-CoV-2 mRNA vaccination elicits robust antibody responses in children

**DOI:** 10.1126/scitranslmed.abn9237

**Published:** 2022-07-26

**Authors:** Yannic C Bartsch, Kerri J St Denis, Paulina Kaplonek, Jaewon Kang, Evan C Lam, Madeleine D Burns, Eva J Farkas, Jameson P Davis, Brittany P Boribong, Andrea G Edlow, Alessio Fasano, Wayne G Shreffler, Dace Zavadska, Marina Johnson, David Goldblatt, Alejandro B Balazs, Lael M Yonker, Galit Alter

**Affiliations:** ^1^ Ragon Institute of MGH, MIT, and Harvard, Cambridge, MA 02139, USA; ^2^ Massachusetts General Hospital Department of Pediatrics, Mucosal Immunology and Biology Research Center, Boston, MA 02114, USA; ^3^ Massachusetts General Hospital Department of Obstetrics and Gynecology, Division of Maternal-Fetal Medicine, Vincent Center for Reproductive Biology, Boston, MA 02114, USA; ^4^ Massachusetts General Hospital Food Allergy Center, Division of Pediatric Allergy and Immunology, Boston, MA 02114, USA; ^5^ Children’s Clinical University Hospital, Riga, LV-1004, Latvia; ^6^ Great Ormond Street Institute of Child Health Biomedical Research Centre, University College London, London WC1N 1EH, UK

## Abstract

Although children have been largely spared from coronavirus disease 2019 (COVID-19), the emergence of severe acute respiratory syndrome coronavirus 2 (SARS-CoV-2) variants of concern (VOC) with increased transmissibility, combined with fluctuating mask mandates and school re-openings, have led to increased infections and disease among children. Thus, there is an urgent need to roll out COVID-19 vaccines to children of all ages. However, whether children respond equivalently to adults to mRNA vaccines and whether dosing will elicit optimal immunity remains unclear. Here we aimed to deeply profile the vaccine-induced humoral immune response in 6 to 11 year old children receiving either a pediatric (50 μg) or adult (100 μg) dose of the mRNA-1273 vaccine and to compare these responses to vaccinated adults, infected children, and children that experienced multisystem inflammatory syndrome in children (MIS-C). Children elicited an IgG-dominant vaccine-induced immune response, surpassing adults at a matched 100 μg dose, but more variable immunity at a 50 μg dose. Irrespective of titer, children generated antibodies with enhanced Fc-receptor binding capacity. Moreover, like adults, children generated cross-VOC humoral immunity, marked by a decline of omicron-specific receptor binding domain-binding, but robustly preserved omicron spike protein-binding. Fc-receptor binding capabilities were also preserved in a dose dependent manner. These data indicate that both the 50 μg and 100 μg doses of mRNA vaccination in children elicits robust cross-VOC antibody responses and that 100 μg doses in children results in highly preserved omicron-specific functional humoral immunity.

## Teaser

SARS-CoV-2 mRNA vaccination elicits robust humoral immune responses in children 6 to 11 years of age.

## INTRODUCTION

The burden of respiratory infections is often higher in young children with a developing, untrained immune system (*1*). However, lower rates of disease were noted early in the coronavirus disease 2019 (COVID-19) pandemic among children, who largely experienced asymptomatic or pauci-symptomatic SARS-CoV-2 infections (*2*). However, with the rise of highly infectious variants of concern (VOCs), like the omicron VOC, increasing infection rates and hospitalization rates for children have been observed globally (*3*, *4*). Linked to the unpredictable incidence of multisystem inflammatory syndrome in children (MIS-C) and the clear contribution children make to population-level spread, the need for vaccines for children is evident (*5*, *6*). However, whether newly emerging COVID-19 vaccine platforms, approved for teenager and adult use, elicit immunity in children is not well understood.

Epidemiologic data clearly highlight vulnerabilities in the pediatric immune system, with increased rates of respiratory, enteric, and parasitic infections disproportionately causing disease in children in the first decade of life (*7*, *8*). In fact, vaccine-induced immune responses often differ across children and adults (*9*). However, whether these vulnerabilities to infection and poor response to protein-based vaccination will translate to newer vaccine platforms, like mRNA vaccine platforms, remains unclear. Moreover, emerging data suggests that dosing may not be straightforward for mRNA vaccines (*10*, *11*), due to reduced immunogenicity in young children, requiring deeper immunologic insights to guide rational pediatric vaccine design.

To begin to define the humoral mRNA vaccine responses in children, we comprehensively profiled vaccine-induced immune responses in children (6 to 11 years) who received the pediatric (50 μg) or adult (100 μg) dose of the mRNA-1273 vaccine regimen, respectively. We observed 100% vaccine response rates prior to the second vaccine dose in children that received the 100 μg vaccine dose. Whereas immune profiles in the low (50 μg) dose were more similar to adults (who received the adults recommended 100 μg dose), children receiving the adult (100 μg) dose generated disproportionately higher IgG biased vaccine responses following the second vaccine dose, with enhanced Fc-effector profiles. Moreover, both pediatric and adult doses elicited broad cross-variant isotype and Fc-receptor binding antibodies; however, although all groups experienced a loss of omicron-receptor binding domain (RBD) reactivity, omicron Spike protein-specific immunity was largely preserved. Further, children receiving the 100 μg dose of the vaccine exhibited the highest cross-reactivity. Collectively, these data point to robust, but dose-dependent, functional humoral pediatric immune signatures induced in children following mRNA-1273 vaccination.

## RESULTS

### Study Cohort

In the wake of fluctuating mask mandates, school re-openings, and the rapid spread of the highly infectious SARS-CoV-2 delta and omicron variants, a surge of SARS-CoV-2 infections in children has been observed (*5*). The numbers of children with severe COVID-19 or life-threatening Multisystem Inflammatory Syndrome in Children (MIS-C) have increased. Because of this, plus our evolving appreciation of children in the spread of the pandemic, there is an urgent need to roll out vaccines across all ages. With the rapid deployment of mRNA vaccines, it remains unclear whether children will generate sufficiently robust immunity following mRNA vaccination. Here we deeply characterized the immune response induced by the Moderna mRNA-1273 vaccine in children that received an adult dose (100 μg) of mRNA-1273 (n=12; median age= 9 years range: 7 to 11 years; 42% female), matching the recommendations for adults, or a pediatric (50 μg) dose of mRNA-1273 (n=12; median age= 8 years range: 6 to 11 years; 50% female) at days 0 and 28, respectively. Plasma samples were collected before vaccination (V0), approximately four weeks after prime (V1) and four weeks after second (V2) immunization (table S1). Both vaccinations were equally well-tolerated between both the 50 μg and the 100 μg doses. Common minor vaccine-related symptoms included pain at injection site, fever, and fatigue (table S2).

### mRNA vaccines induce robust SARS-CoV-2 spike protein-binding and neutralizing titers in children.

**Fig. 1.**
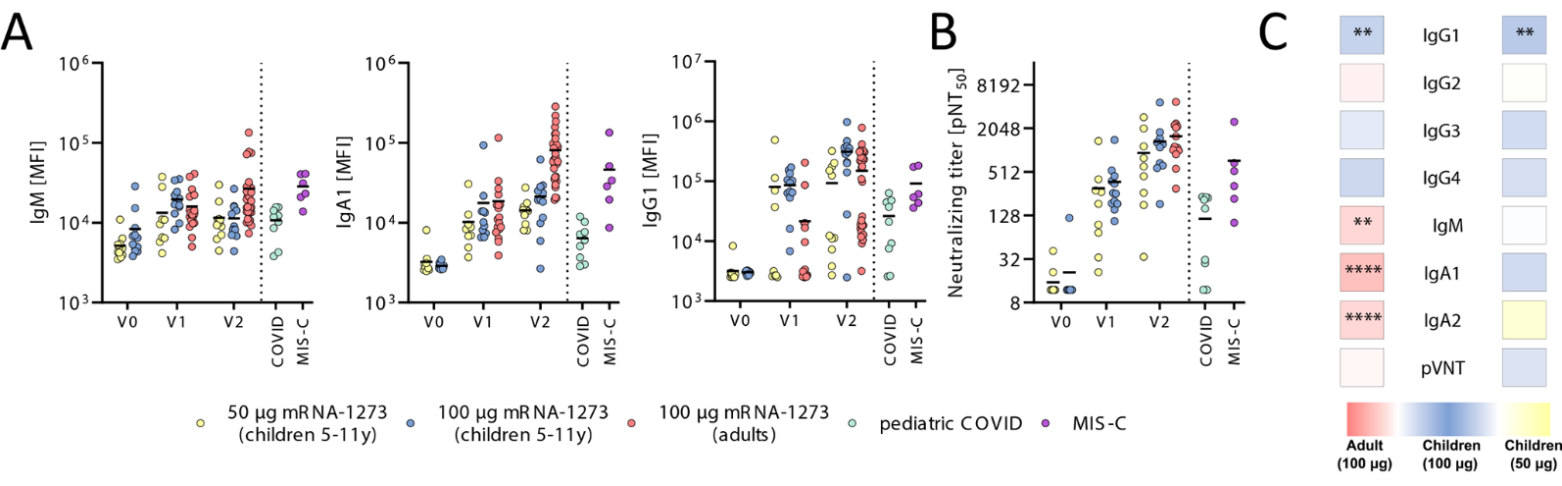
mRNA-1273 vaccination induces robust binding and neutralizing titers in children. (**A**) Relative SARS-CoV-2 spike protein (Wuhan)-specific IgM, IgA1 and IgG1 binding was determined by Luminex in children (6 to 11 years) receiving 50 μg or 100 μg mRNA1273 before (V0_50 μg_: n=12; V0_100 μg_: n=12), after the first (V1_50 μg_: n=9; V1_100 μg_: n=12) or after the second (V1_50 μg_: n=11; V2_100 μg:_ n=12) dose or in adults receiving two 100 μg doses (V1: n=19; V2: n=33) as well as in samples from individuals after pediatric COVID (n=9) or MIS-C (n=6). MFI, median fluorescence intensity. (**B**) The dot plots show the inverse 50% pseudovirus neutralizing titers (pNT_50_) in children (6-11 years) receiving 50 μg or 100 μg mRNA1273 before (V0_50 μg_: n=12; V0_100 μg_: n=12), after the first (V1_50 μg_: n=9; V1_100 μg_: n=12) or after the second (V1_50 μg_: n=9; V2_100 μg:_ n=11) dose or in adults receiving two 100 μg doses (V2: n=14) as well as in convalescent pediatric COVID (n=9) or MIS-C (n=6) samples. Horizontal bars in (A) and (B) indicate mean. (**C**) Heatmap strips summarize univariate comparison at the V2 timepoint of 100 μg dose vaccinated children to adults (left panel) or to 50 μg dose vaccinated children (right panel). pVNT, pseudovirus-neutralizing titer. The color of the tiles indicate whether antibody binding titer were up-regulated in the respective group: 100 μg vaccinated children (blue shades), adults (red shades), or 50 μg vaccinated children (yellow shades). A Wilcoxon-signed rank test was used to test for statistical significance and asterisks indicate statistically significant differences of the respective feature after Benjamini-Hochberg correction for multiple testing (**p<0.01;***p<0.001; ****p<0.0001).

To begin to investigate the vaccine-induced humoral response, we profiled SARS-CoV-2 spike protein-specific antibody titers. At V1, we observed seroconversion (marked by an increase in spike protein-specific IgM, IgA1 or IgG1 binding compared to V0) in 100% of children receiving the 50 μg (n=9/9) or 100 μg (n=12/12) dose of mRNA-1273 (**Fig. 1A**) with comparable neutralizing antibody titers in the vaccinated pediatric and adult cohorts (**Fig. 1B**). Both spike protein-specific IgA1 and IgG1 increased with the second dose, whereas spike protein-specific IgM responses declined slightly, marking efficient class switching. After the second dose we observed significantly elevated spike protein-specific IgA1 concentrations in adults compared to children (p < 0.001) (**Fig. 1C**) . In contrast, children in the 100 μg dose group elicited higher IgG1 titers after the first and second dose of the vaccine compared to children in the 50 μg dose group (p = 0.004), as well as compared to vaccinated adults (p = 0.03) (**Fig. 1C**). Univariate comparison of V2 abundance of the 100 μg adults to 100 μg and 50 μg children highlighted isotype selection differences across children and adults, but minimal overall differences in antibody binding titers and neutralization across the 50 μg and 100 μg doses in children (**Fig. 1B** **and** **1C**). Furthermore, vaccine-induced binding and neutralization titers in the 100 μg pediatric dose group were higher compared to those observed in naturally exposed convalescent children with COVID-19 or acute MIS-C which required hospitalization and ICU treatment. Importantly, although these differences may be related to exposure to different variants, we observed superior vaccine-induced binding to all variants, highlighting the critical importance of SARS-CoV-2 vaccination in promoting broader VOC immunity in children compared to infection (**fig. S1**). Taken together, these data show that mRNA vaccination can elicit strong but dose-dependent anti-SARS-CoV-2 binding and neutralizing titers in children superior to natural infection that are accompanied by some age-dependent shifts in isotype-antibody profiles.

### mRNA vaccination induces highly potent spike protein-specific Fc effector functions in children.

**Fig. 2.**
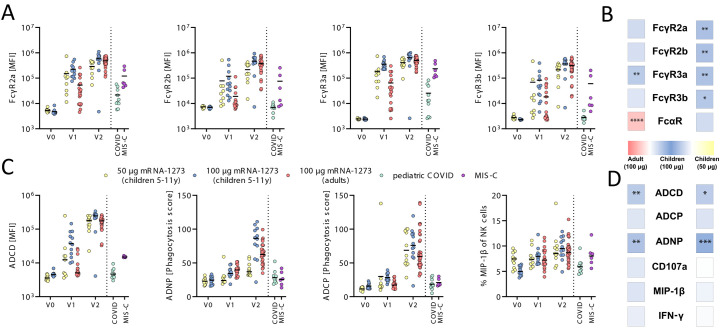
mRNA-1273 vaccination induces higher FcγR binding and phagocytic activity in children. (**A**) Binding of SARS-CoV-2-specific antibodies to FcγR2a, 2b, 3a, and 3b was determined by Luminex in children (6 to 11 years) receiving 50 μg or 100 μg mRNA1273 before (V0_50 μg_: n=12; V0_100 μg_: n=12), after the first (V1_50 μg_: n=9; V1_100_: n=12) or after the second (V2_50 μg_: n=11; V2_100 μg:_ n=12) dose or in adults receiving two 100 μg doses (V1: n=19; V2: n=33) as well as in convalescent pediatric COVID (n=9) or MIS-C (n=6) samples. (**B**) Heatmap strips summarize univariate comparison of Fc-receptor binding at the V2 timepoint of 100 μg dose vaccinated children to adults (left panel) or to 50 μg dose vaccinated children (right panel). Color of the tiles indicate whether antibody binding titer were up-regulated in the respective group: 100 μg vaccinated children (blue shades), adults (red shades), or 50 μg vaccinated children (yellow shades). (**C**) The ability of SARS-CoV-2 spike protein-specific antibody Fc to induce ADCD, ADNP, cellular THP-1 monocyte phagocytosis (ADCP), or activation of NK cells marked by expression of MIP-1β was analyzed in the same samples as in (A). (**D**) Heatmap strips summarize univariate comparison of Fc effector functions at the V2 timepoint of 100 μg dose vaccinated children to adults (left panel) or to 50 μg dose vaccinated children (right panel) as in (**B**). A Wilcoxon-signed rank test was used to test for statistical significance in (C) and (D) and asterisks indicate statistically significant differences of the respective feature after Benjamini-Hochberg correction for multiple testing (*p<0.05; **p<0.01;***p<0.001; ****p<0.0001).

In addition to binding and neutralization, protection against severe adult COVID-19 has been linked to the ability of antibodies to leverage additional antiviral functions by Fc-receptors to fight infection, referred to as antibody effector functions (*12*–*14*). Specifically, opsonophagocytic pathogen clearance is key to protection against several bacterial pathogens and cytotoxic antibody functions have been linked to protection against viruses (*15*, *16*). Thus, we profiled the relative ability of vaccine-induced immune responses to bind to human Fc-receptors (FcγR2a, FcγR2b, FcγR3a, FcγR3b, and FcαR) as well as their ability to elicit antibody-dependent complement deposition (ADCD), antibody-dependent neutrophil phagocytosis (ADNP), antibody-dependent monocyte phagocytosis (ADCP), or antibody-dependent natural killer cell activation (ADNKA). Children in both dose groups elicited spike protein-specific IgG antibodies that bound robustly to all Fc-receptors following the first dose, greater than responses observed in vaccinated adults and in natural COVID-19 infection or MIS-C (**Fig. 2A**). Moreover, these responses expanded further after the second immunization resulting in significantly elevated antibody-Fc-receptor binding in the 100 μg pediatric dose group compared to the 50 μg dose (p_FcγR2a_ = 0.001, p_FcγR2b_<0.001, p_FcγR3a_=0.002 and p_FcγR3b_=0.004, respectively), with significantly higher FcγR3a (p=0.004) but otherwise similar FcγR2a, FcγR2b and FcγR3b binding in the 100 μg pediatric dose group as compared to the 100 μg adult group (**Fig. 2B**). This increased Fc-receptor binding was not directly related to overall changes in spike protein-specific IgG subclass selection (**fig. S1**), pointing to alternate mechanisms for augmented humoral immune function in children, potentially linked to pediatric selection of more potent Fc-glycosylation profiles (*17*). In contrast, compared to adults, children induced lower concentrations of IgA antibodies that exhibited, as expected, lower interactions with the IgA-Fc-receptor, FcαR, compared to adults (**Fig. 2B** **and fig. S2A**) (*18*, *19*).

To next determine whether these distinct pediatric Fcγ-receptor binding profiles translated to more functional spike protein-specific humoral immune responses, we examined vaccine-induced Fc-effector functions (**Fig. 2C**). Low degrees of ADCD, ADNP and ACDP were observed after primary immunization, but were augmented by the second immunization across the groups, resulting in significantly increased ADCD and ADNP in the 100 μg vaccinated children compared to adults or the 50 μg pediatric dose (**Fig. 2C** **and** **2D**; p_ADCD_=0.007 and p_ADNP_<0.001). In contrast, NK cell functions (as measured by macrophage inflammatory protein (MIP)-1β, interferon gamma (IFNγ) and CD107a expression) were induced to equal degrees across all groups (**Fig. 2D** **, fig. S2B**). Overall, high dose mRNA-1273 induced a higher abundance of ADCD- and ADNP-promoting antibodies in children compared to adults following the 100 μg vaccine regimen (**Fig. 2D**). These data point to enhanced functional antibody responsiveness in children at a matched 100 μg dose compared to adults, and a solid functional response at the optimized 50 μg dose, endowing children with a robust functional immune response at half the adult dose, all of which were to a higher degree than those observed following natural infection or MIS-C.

### mRNA vaccination in children results in selective expansion of opsonophagocytic antibodies.

**Fig. 3.**
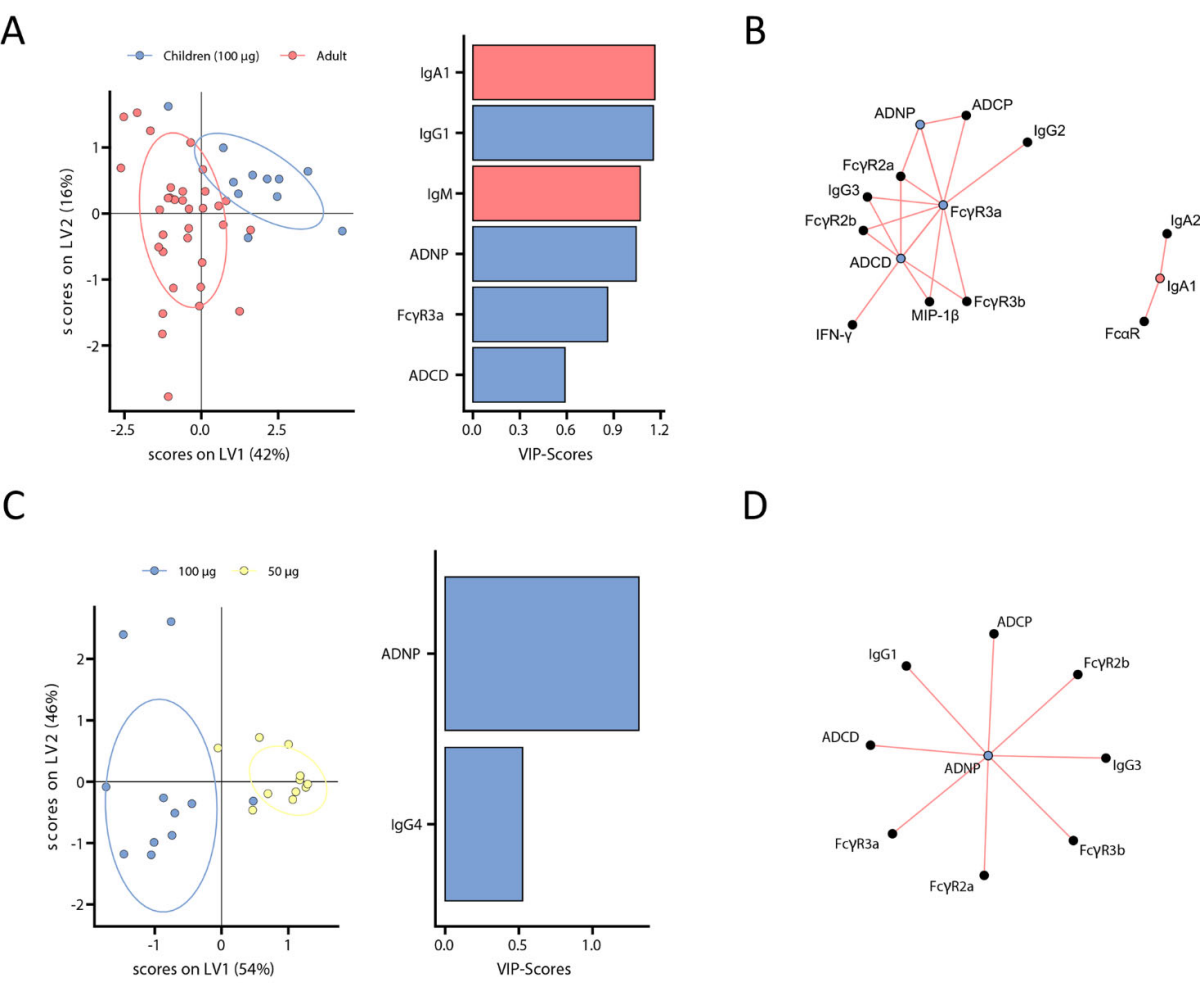
Distinct humoral profiles distinguish adult and pediatric vaccine responses. (**A**) A machine learning model was built using a minimal set of LASSO-selected SARS-CoV-2 spike protein-specific features at V2 (left panel) to discriminate between vaccine responses in adult (red) and 100 μg vaccinated children (purple) in a PLS-DA analysis (right panel, VIP indicates the Variable Importance Projection Score). (**B**) A co-correlation network illustrates all LASSO-selected features. Nodes of selected features are colored whether they were enriched in children (purple) or adults (red). Lines indicate significant (p<0.05) spearman correlations with |r|>0.7 of connected features (only positive correlations with r>0 were observed). (**C**) PLS-DA model of LASSO selected features at V2 (left panel) to discriminate between vaccine responses in 100 μg (purple) and 50 μg (yellow) vaccinated children. (**D**) A co-correlation network as in (B) illustrates all LASSO-selected features. A single node of a selected features is colored in purple to indicate enrichment in samples from 100 μg vaccinated children.

To gain a more granular sense of the differences in immune responses across children and adults at matched doses or across children receiving the 50 μg and 100 μg doses, we next utilized a machine learning approach to probe the humoral immune features that differed most across these groups. As few as six of the overall features analyzed across all plasma samples were sufficient to completely resolve vaccine-induced immune responses induced by the 100 μg dose across children and adults (**Fig. 3A and B**). Specifically, vaccine-induced spike protein-specific IgG1, FcγR3a binding, ADNP, and ADCD were all enriched selectively in children, whereas spike protein-specific IgM and IgA1 titers were enriched in adults (**Fig. 3A**), highlighting distinct isotype selection in adults, and the generation of more functional antibodies in children. Conversely, comparison of 50 μg and 100 μg doses in children was achieved using only two of all antibody features analyzed for each plasma sample. These features spike protein-specific ADNP and IgG4 concentrations, both of which were enriched in the immune profiles in children that received the 100 μg dose of the vaccine (**Fig. 3C and D**). Additionally, in contrast to infection, vaccination induced higher titers and higher degrees of antibody function in vaccinated children to those that were previously infected (**Fig. 2** **and fig. S3**). Collectively, these data point to slight shifts in isotype selection between adults and children, but the potential for children to raise more antibodies with effector functions.

### mRNA vaccination in children raises robust responses against SARS-CoV-2 VOCs.

**Fig. 4.**
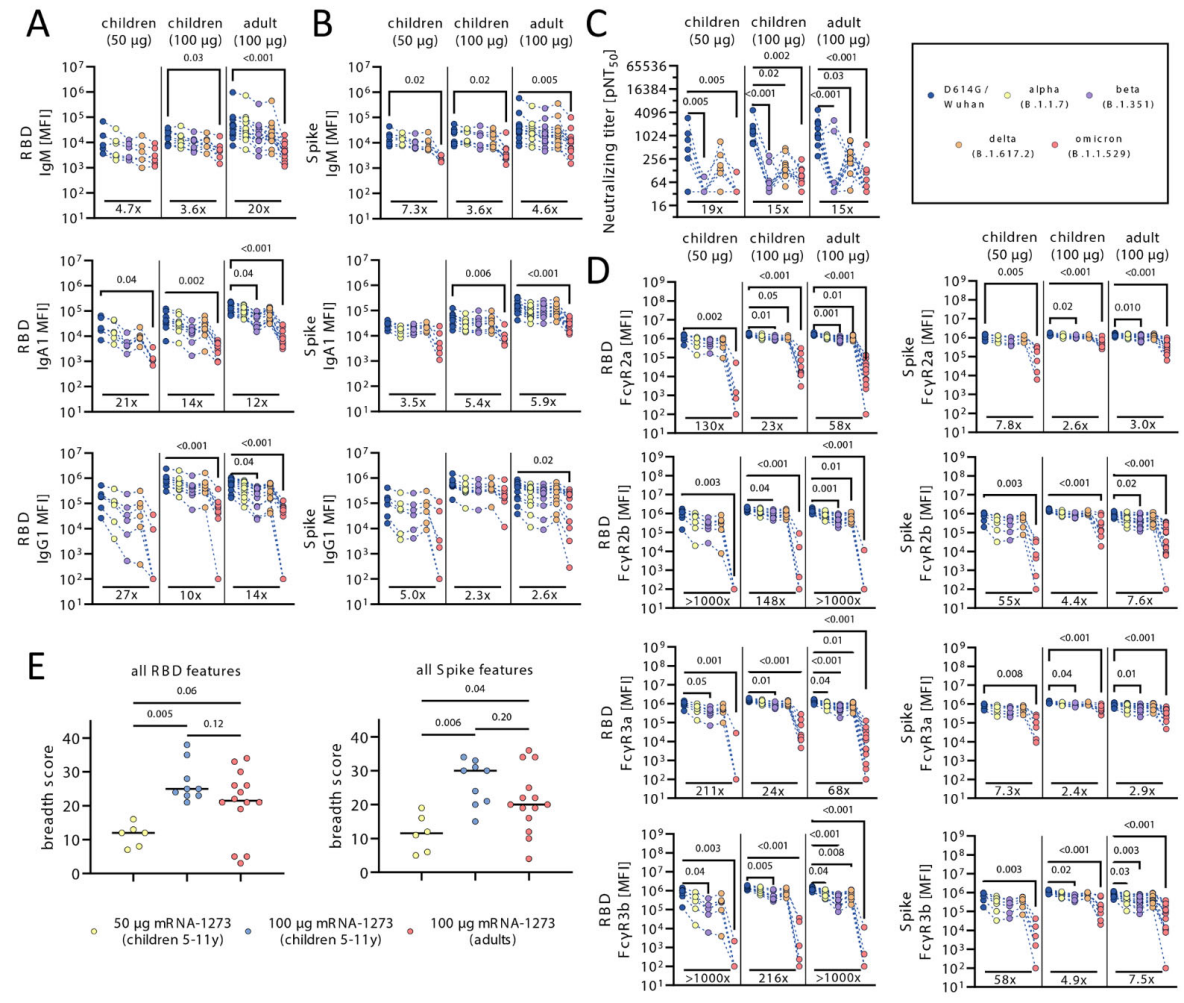
mRNA-1273 vaccination elicits humoral responses to SARS-CoV-2 VOCs. (**A and B**) The line graphs show the vaccine-induced IgM, IgA1 and IgG1 recognition to D614G (wild-type; blue), alpha (B.1.1.7; yellow), beta (B.1.351; purple), delta (B.1.617.2; orange), and omicron (B.1.1.529; red) variants of concern RBDs (A) or full spike protein (B). (**C**) neutralizing titers are shown as pNT_50_ values. All plots in (A to C) show results for samples from children (n_50 μg_=6, n_100 μg_=9) and adults (n=14) at V2, where each individual’s response is linked across VOC antigens. (**D**) The line graphs show the FcγR (FcγR2a, FcγR2b, FcγR3a, FcγR3b) binding profiles of vaccine-induced antibodies to RBD or spike protein VOC antigens across children (n_50μg_=6, n_100μg_=9) or adults (n=14) at V2, where each individual’s response is linked across VOC antigens. (E) Breadth score was calculated by summing up the number of VOC RBD- or spike protein-specific features above the median value for each individual (n_50 μg_=6, n_100 μg_=9) or adults (n=14) at V2. Horizontal bars in (E) indicate median. Background corrected data are shown and negative values were set to 100 for graphing purposes in (A to D). A Kruskal-Wallis test with a Benjamini-Hochberg post-test correction for multiple comparisons was used to test for statistical differences between wild-type and VOC titers within groups in (A to D) or between the groups in (E). P-values for significantly different features are shown above and fold change reductions of omicron titers compared to wild-type are shown below each dataset. Data for adult samples has been partly published previously (*55*)).

Real-world effectiveness data suggest that mRNA vaccines confer robust protection against severe disease and death against the original (wild-type) SARS-CoV-2 strain (Wuhan) at greater than 90% (*20*). This degree of effectiveness appears to be sustained against evolving VOCs, including the alpha and delta variants, although lower degrees of protection have been observed against the beta variant (*21*, *22*). Whereas previous VOCs were marked by single or few amino acid substitutions, the omicron variant (B.1.1.529 or BA.1) has 29 mutations in the spike protein, resulting in enhanced transmissibility and a concomitant loss of neutralizing titers (*23*, *24*). Yet, despite the striking increase in omicron transmissibility, a similar increase in severe disease and death has not been observed, suggesting that alternate vaccine-induced immune responses may continue to afford protection against severe disease and death. We thus explored whether mRNA vaccination in children resulted in the generation of responses with differential VOC recognition capabilities (*25*). We observed a progressive loss of IgM, IgA, and IgG binding to VOC RBDs across both pediatric groups and adults, with more variable cross-VOC IgG responses among 50 μg immunized children, but a consistent and substantial loss of binding to the omicron RBD across all 3 groups (**Fig. 4A**). Conversely spike protein-specific responses were more resilient across most VOCs and across the 3 groups, except for omicron spike protein-specific responses that were considerably lower across IgM and IgA response across the groups (**Fig. 4B**). Yet, IgG responses showed 3 different patterns: 1) 50 μg immunized children experienced heterogeneous responses across VOCs, marked by some of the lowest omicron specific-responses, 2) adults exhibited more stable variant spike protein-binding IgG concentrations, but experienced a substantial reduction in omicron variant spike protein reactivity, and 3) 100 μg immunized children exhibited the least reduction in spike protein-specific recognition across VOCs, including to the omicron variant spike protein. Along these lines, neutralization, which mostly relies on epitopes within the RBD, was reduced to different VOCs pseudoviruses compared to the wildtype vaccine insert across the groups (**Fig. 4C**). Furthermore, Fc-receptor binding capability was largely preserved across RBD VOCs, except for omicron RBD-binding, which was substantially lower across all 3 groups (**Fig. 4D**). However, the degree of binding to VOC spike proteins differed across the 3 groups. Whereas wild-type, alpha, beta, and delta VOC Fc-receptor binding profiles were highly preserved across all 3 groups, omicron variant spike protein-specific Fc-receptor binding was most affected in samples from 50 μg immunized children (**Fig. 4D**). Adults exhibited an intermediate loss of Fc-receptor binding antibodies specific to the omicron spike protein, with some preservation of the opsonophagocytic FcγR2a and cytotoxic FcγR3a binding. Conversely, 100 μg immunized children exhibited the smallest degree of loss of Fc-receptor binding to the omicron spike protein, suggesting the presence of highly resilient antibodies in children receiving the adult dose that continue to bind to the omicron spike protein, despite the loss of RBD binding. Finally, to analyze whether children generate broader or more flexible humoral immune response at the 100 μg dose, enabling them to preserve immunity to VOCs, we calculated a RBD or spike protein breadth score by summing up the number of VOC features with a value higher than the median across the population (**Fig. 4E** **and fig. S4**). Children that received the 100 μg dose exhibited the highest breadth score for RBD and spike protein, indicating increased recognition of different VOCs. Conversely, children that received the 50 μg dose exhibited significantly reduced breadth compared to children in the 100 μg dose (**Fig. 4E**; RBD features, p=0.005; spike features, p=0.006), pointing to qualitative difference across the doses. Moreover, to probe the impact of pre-existing coronavirus immunity, responses were compared across groups to other circulating human coronaviruses (**fig. S5**). Pre-existing coronavirus titers were associated with augmented vaccine response after each dose, pointing to limited evidence of original antigenic sin. Whether differences in VOC recognition, SARS-CoV-2 neutralization, and Fc-function lead to differences in disease breakthrough across the ages remains unclear but provides some additional immunological insights that may continue to explain the epidemiologic differences in disease severity in the setting of emerging VOCs.

## DISCUSSION

Immunity elicited by mRNA vaccine platforms responds rapidly to SARS-CoV-2 infection, demonstrating high degrees of effectiveness in adult populations (*26*, *27*). However, despite the successes of SARS-CoV-2 vaccines, the global roll out of these vaccines has begun to highlight key vulnerable populations and strategic gaps that may limit the impact of vaccination globally. Although children generally experience mild symptoms, they can harbor high concentrations of SARS-CoV-2 virions, thereby contributing substantially to viral spread (*28*–*30*). Furthermore, increasing numbers of children are suffering from severe COVID-19, with over 43,000 hospitalizations and over 1,000 deaths in the US alone as of June 2022 (*31*). However, because children have a more naïve immune system that evolves with age, it was uncertain how the mRNA vaccine platforms would impact immunogenicity in young children. Additionally, recent results suggest that dose adjustments for very young children, due to safety and tolerance concerns, have introduced additional variation in immunogenicity, resulting in poor immunogenicity in children under 5 years who received a lower dose than the adult recommended dose (*11*). Thus, in the absence of empirical data, optimal dosing is uncertain; here we aimed to dive deeply in defining humoral profile differences across doses and across children and adults. Similar to results with the Pfizer and Moderna mRNA vaccine trials in teenagers (*32*, *33*), here we found that the Moderna mRNA vaccine was highly immunogenic in 6 to 11 year old children, generating a humoral response superior to that seen following viral exposure. However, granular vaccine-induced humoral profiling identified differences in adult and pediatric vaccine responses, marked by a selective induction of highly functional IgG responses, with fewer IgA and IgM responses compared to adults. At a matched 100 μg dose, children mounted robust opsonophagocytic functions and Fcγ-receptor binding responses. Moreover, at half the adult dose, children mounted equal, albeit more variable, responses compared to adults. Although both doses were equally well tolerated in children in our cohort, concerns of serious adverse events, including myocarditis (*34*), have been reported in adolescents receiving adult dosing of the BNT162b2 mRNA vaccination. It has been postulated that lower mRNA vaccine doses in children could reduce the likelihood of adverse events; however it remains elusive whether the humoral response induced by the 50 μg dose may be accompanied by a more variable degree of protection. Yet, early data from BNT162b2-immunized children suggests that lower doses in children confer robust protection against disease (*35*).

Virus neutralization represents a key surrogate marker of vaccine protection against COVID-19. Yet despite the loss of neutralization against several emerging variants of concern (VOCs), mRNA vaccines continue to provide protection against severe disease and death (*21*, *36*). Interestingly, opsonophagocytic functions of antibodies, rather than neutralization alone, have been linked to survival of COVID-19 following infection (*12*) and are associated with protection from infection in animal models (*37*, *38*). Here, when immunized with the adult dose, children developed comparable neutralization profiles, but exhibited a preferential expansion of opsonophagocytic functions compared to adults. The enhanced opsonophagocytic function was not linked to differential subclass or isotype selection, suggesting that children may induce more functional antibodies through alternate changes to the humoral immune response, including potential differences in post-translational IgG modification, that may lead to more flexible, highly functional responses, representing an evolutionary adaptation enabling children to react more adaptability to infections (*39*).

The adaptive immune response matures during the first decade of life (*40*). Several lines of evidence suggest that the more naïve immune response in children may allow the immune system to evolve more easily to pathogens, making it poised to generate broader immunity to new viruses (*41*, *42*). Moreover, throughout life, our naïve clonal repertoire or immune cells shift in response to the sequence of pathogens and vaccines to which we are exposed. Thus, naïve children may have a less “biased” repertoire, enabling the generation of immunity to a broader range of pathogens (*43*). Along these lines, we observed robust induction of immunity against most VOCs, with the exception of the omicron variant. IgG and Fc-receptor binding profiles were highly similar among children and adults, although children immunized with the 100 μg dose induced IgG that exhibited more recognition of the omicron spike protein in comparison to children immunized with the 50 μg dose and 100 μg immunized adults. Likewise, children in the 100 μg dose group elicited responses with an increased cross-VOC breadth, whereas children in the 50 μg dose exhibited reduced cross-VOC recognition. These data suggest that higher pediatric dosing can result in more flexible humoral immunity in children against highly divergent VOCs, potentially superior to those induced in adults at a matched dose. Thus, at the right dose, children may generate more functional Fc-effector functions, that, although not neutralizing, may be poised for rapid elimination of the pathogen upon transmission, providing a highly effective means to prevent COVID-19 in this population.

Our study does have limitations. We were limited in the number of pediatric samples collected and analyzed, but differences were still noted in the humoral immune response after vaccination and infection. Timing of sample collection represents another limitation. We analyzed vaccine antibody-responses in children for up to 4 weeks after the second dose, when titers were still high. It will be critical to analyze durability of these response and to determine whether these responses will wane differentially across doses and ages, whether they will be more protective against particular VOCs, and whether children will require booster doses of vaccine.

With the increasing spread of SARS-CoV-2 VOCs (*44*), increasing incidence of COVID-19 among the pediatric population, the rare but serious development of MIS-C, and the recent appreciation for long-COVID in children, the need to determine whether SARS-CoV-2 vaccines can elicit functional immune responses will be key to protect children (*3*, *5*, *28*, *45*, *46*). Comparable to previous observations in adults (*10*), the mRNA-1273 vaccine induced robust binding titers, neutralization, and Fc effector functions in vaccinated children in a dose dependent manner; these responses were often higher than those observed in children diagnosed with COVID-19 or MIS-C, pointing to the importance of vaccination to robustly bolster immunity to SARS-CoV-2. Together, these findings support vaccination of children with mRNA-1273 as a safe and effective strategy to protect children against COVID-19, MIS-C, and long-COVID.

## MATERIALS AND METHODS

### Study Design

To compare antibody responses elicited by the Moderna SARS-CoV-2 vaccine mRNA-1273, pediatric vaccine samples were obtained from children who were vaccinated with two doses of 100 μg of the mRNA-1273 vaccine or two doses of 50 μg of the mRNA-1273 in a Phase2/3 clinical trial (ClinicalTrials.gov Identifier: NCT04796896) at Massachusetts General Hospital. Additionally, we included samples from nine children at convalescence (median duration since symptom onset: 52 days) who previously presented with polymerase chain reaction (PCR)-confirmed COVID-19 (age range 1 to 9 years) and six children with acute MIS-C (age range 3 to 21 years) (table S1). Samples from 33 adults (age range 20 to 55 years) who received two doses of 100 μg of the mRNA-1273 were also included as controls (ClinicalTrials.gov Identifiers: NCT04380896 and NCT04283461). All pediatric participants provided informed assent, when age appropriate, and participants or their legal guardian provided informed consent prior to participation. Blood samples were collected prior to vaccination (V0), approximately one month after the first vaccination (V1) and one month after the second vaccination (V2). This study was overseen and approved by the MassGeneral Brigham Institutional Review Board (IRB #2020P00955). Buffy coats for NK cell preparation from healthy volunteers were collected and processed by the Massachusetts General Hospital (MGH) Blood Bank. Whole blood samples for neutrophil isolation were collected at the Ragon Institute of MGH, MIT and Harvard. All donors were 18 years or older and gave signed consent. Samples were deidentified before use, and the study was approved by the MGH Institutional Review Board.

### Antigens and biotinylation

SARS-CoV-2 D614G or variants of concern Spike and RDB proteins of were expressed in mammalian HEK293 cells and obtained from SinoBiological. All antigens were biotinylated using an NHS-Sulfo-LC-LC kit according to the manufacturer’s instruction (Thermo Fisher Scientific). If required by the assay, excessive biotin was removed by size exclusion chromatography using Zeba-Spin desalting columns (7kDa cutoff, Thermo Fisher Scientific).

### Antibody isotype and Fc-receptor binding

Antigen-specific antibody isotype and subclass titers and Fc-receptor binding profiles were analyzed with a custom multiplex Luminex assay as described previously (*47*). In brief, antigens were coupled directly to Luminex microspheres (Luminex Corp). Coupled beads were incubated with diluted plasma samples washed, and Ig isotypes or subclasses with a 1:100 diluted phycoerythrin (PE)-conjugated secondary antibody (anti-human IgG1 (Cat# 9052-09, RRID:AB_2796621), IgG2 (Cat# 9060-09, RRID:AB_2796635), IgG3 (Cat# 9210-09, RRID:AB_2796701), IgG4 (Cat# 9200-09, RRID:AB_2796693), IgM (Cat# 9020-09, RRID:AB_2796577), IgA1 (Cat# 9130-09, RRID:AB_2796656) or IgA2 (Cat# 9140-04, RRID:AB_ 2796661) all from Southern Biotech). For the FcγR binding, a respective PE–streptavidin (Agilent Technologies) coupled recombinant and biotinylated human FcγR protein was used as a secondary probe. Excessive secondary reagent was washed away after a 1 hour incubation, and the relative antigen-specific antibody concentrations were determined on an iQue analyzer (Intellicyt). Each sample was analyzed in duplicate.

### Pseudovirus neutralization assay

Three-fold serial dilutions were performed for each plasma sample before adding 50 to 250 infectious units of pseudovirus expressing the SARS-CoV-2 reference (Wuhan/wild-type), beta, delta or omicron variant spike protein to human angiotensin converting enzyme 2 (hACE2) expressing HEK293 cells for 1 hour. Dilutions ranged from 1:12 to 1:8748 for wild-type and delta and 1:36 and 1:8748 for beta and omicron due to limited sample volume. Percentage neutralization was determined by subtracting background luminescence measured in cell control wells (cells only) from sample wells and dividing by virus control wells (virus and cells only). Pseudovirus neutralization titers (pNT_50_) values were calculated by taking the inverse of the 50% inhibitory concentration value for all samples with a pseudovirus neutralization value of 80% or higher at the highest concentration of serum.

### ADCD assay

Complement deposition was performed as described before (*48*). In brief, biotinylated antigens were coupled to FluoSphere NeutrAvidin beads (Thermo Fisher Scientific) and, to form immune complexes, incubated with 10 μl 1:10 diluted plasma samples for 2 hours at 37°C. After non-specific antibodies were washed away, immune-complexes were incubated with guinea pig complement in GVB++ buffer (Boston BioProducts) for 20 min at 37°C. EDTA-containing phosphate-buffered saline (15mM) was used to stop the complement reaction and deposited C3 on beads was stained with anti-guinea pig C3-fluroescein isothiocyanate (FITC) antibody (MP Bio Cat# 0855385, RRID:AB_2334913, 1:100) and analyzed on an iQue analyzer (Intellicyt). Each sample was analyzed in duplicate.

### ADNP assay

Phagocytosis score of primary human neutrophils was determined as described before (*49*). Biotinylated antigens were coupled to FluoSphere NeutrAvidin beads (Thermo Fisher Scientific) and incubated with 10 μl 1:100 diluted plasma for 2 hours at 37°C to form immune complexes. Primary neutrophils were derived from Ammonium-Chloride-Potassium (ACK) buffer lysed whole blood from healthy donors (see above) and incubated with washed immune complexes for 1 hour at 37°C. Afterwards, neutrophils were stained for surface CD66b (BioLegend Cat# 305111, RRID:AB_2563293, Pacific Blue conjugated) expression, fixed with 4% paraformaldehyde and analyzed on a iQue analyzer (Intellicyt). Each sample was analyzed in duplicate using neutrophils from two different blood donors (biological duplicate).

### ADCP assay

THP-1 phagocytosis assay was performed as described before (*50*). In brief, biotinylated antigens were coupled to FluoSphere NeutrAvidin beads (Thermo Fisher Scientific) and incubated with 10 μl 1:100 diluted plasma for 2 hours at 37°C to form immune complexes. THP-1 monocytes (American Type Culture Collection) were added to the beads, incubated for 16 hours at 37°C, and fixed with 4% paraformaldehyde. Samples were analyzed on a iQue analyzer (Intellicyt). Each sample was analyzed in duplicate.

### ADNKA assay

To determine antibody-dependent NK cell activation, MaxiSorp enzyme-linked immunosorbent assay (ELISA) plates (Thermo Fisher Scientific) were coated with respective antigen for 2 hours at room temperature and then blocked with 5% bovine serum albumin (BSA, Sigma-Aldrich). Fifty μl 1:50 diluted plasma sample was added to the wells and incubated overnight at 4°C. NK cells were isolated from buffy coats from healthy donors (see above) using the RosetteSep NK cell enrichment kit (STEMCELL Technologies) and stimulated with recombinant human interleukin-15 (1ng/ml, STEMCELL Technologies) at 37°C overnight. NK cells were added to the washed ELISA plate and incubated together with anti-human CD107a Brilliant Violet (BV)605 (BioLegend Cat# 328634, RRID:AB_2563851, 1:40), brefeldin A (Sigma-Aldrich), and monensin (BD Biosciences) for 5 hours at 37°C. Next, cells were surface stained for CD56 (BD Biosciences Cat# 335791, RRID:AB_399970, 1:200, phycoerythrin (PE) cyanine (cy)-7 conjugated), and CD3 (BioLegend Cat# 300426, RRID:AB_830755, 1:800, allophycocyanin (APC)-cy7 conjugated). After fixation and permeabilization with FIX & PERM Cell Permeabilization Kit (Thermo Fisher Scientific), cells were stained for intracellular markers MIP-1β (BD Biosciences Cat# 562900, RRID:AB_2737877, 1:50, BV421 conjugated) and interferon (IFN)-γ (BD Biosciences Cat# 554701, RRID:AB_395518, 1:17, PE conjugated). NK cells were defined as CD3^-^CD16^+^CD56^+^ and frequencies of degranulated (CD107a^+^), IFN-γ^+^ and MIP-1β^+^ NK cells determined on an iQue analyzer (Intellicyt) (*51*). Each samples was tested with NK cells from three different donors (biological triplicates).

### Calculation of VOC breadth

To calculate the breadth of binding across VOCs, isotype, subclass or Fc-receptor features of the VOCs were normalized to the respective D614G feature to account for age- or vaccine-related differences. For each feature, the median value was calculated and subtracted from the individual values. Each individual with a positive difference (above or at the median) was assigned a 1 and negative difference (below the median) a 0. The feature breadth score per individual was defined as the sum of this matrix for all analyzed VOCs per spike protein- or RBD-specific feature, respectively. A maximum breadth score of 4 was possible if median was higher for all analyzed VOCs for the respective feature. The sum of all breadth scores per spike protein or RBD antigen was plotted in **Fig. 4E**.

### Statistical analysis

Data analysis was performed using GraphPad Prism (v.9.2.0) and RStudio (v.1.3 and R v.4.0). No data points were omitted from the analysis. We assumed non-normal distribution. Comparisons between the adults and children were performed using Wilcoxon-signed rank test followed by Benjamini-Hochberg (BH) correction. A Kruskal-Wallis test with BH correction was performed for the comparison of variant of concern titers. Association of pre-existing coronavirus (strains OC43, NL63, or HKU1) IgG1 titers and SARS-CoV-2 vaccine induced titers were assessed by Spearman correlation.

Multivariate classification models were built to discriminate humoral profiles between vaccination arms. Prior to analysis, all data were normalized using z-scoring. Feature selection was performed using least absolute shrinkage and selection operator (LASSO). Classification and visualization were performed using partial least square discriminant analysis (PLS-DA). Model accuracy was assessed using ten-fold cross-validation. These analyses were performed using R package “ropls” version 1.20.0 (*52*) and “glmnet” version 4.0.2 (*53*). Co-correlates of LASSO selected features were calculated to find features that can equally contribute to discriminating vaccination arms. Correlations were performed using Spearman method followed by Benjamini-Hochberg correction. The co-correlate network was generated using R package “network” version 1.16.0 (*54*). Raw, individual-level data are presented in data file S1.

## Supplementary Materials

### This PDF file includes:

Figs. S1 to S5 Tables s1 and S2

### Other Supplementary Material for this manuscript includes the following:

MDAR Reproducibility Checklist
