# Supplementary material for: SARS-CoV-2 mRNA vaccination elicits robust antibody responses in children
Source: Sci Transl Med. 2022 Jul 26:eabn9237. doi: 10.1126/scitranslmed.abn9237 (PMC9348753; doi:10.1126/scitranslmed.abn9237)
Supplement: Supplementary file 1 — Figs. S1 to S5 Tables s1 and S2 [file scitranslmed.abn9237_sm.pdf]

Science Translational Medicine
AAAS

Supplementary Materials for

SARS-CoV-2 mRNA vaccination elicits robust antibody responses in children

Yannic C Bartsch *et al.*

Corresponding authors: Lael M Yonker, LYONKER@mgh.harvard.edu; Galit Alter, GALTER@mgh.harvard.edu

DOI: 10.1126/scitranslmed.abn9237

The PDF file includes:

Figs. S1 to S5
Tables S1 and S2

Other Supplementary Material for this manuscript includes the following:

MDAR Reproducibility Checklist
Data file S1

SARS-CoV-2 mRNA vaccination elicits robust antibody responses in children

Authors: Yannic C Bartsch[1], Kerri J St Denis[1], Paulina Kaplonek[1], Jaewon Kang[1], Evan C Lam[1], Madeleine D Burns[2], Eva J Farkas[2], Jameson P Davis[2], Brittany P Boribong [2], Andrea G Edlow[3], Alessio Fasano[2], Wayne G Shreffler[4], Dace Zavadska[5], Marina Johnson[6], David Goldblatt[6], Alejandro B Balazs[1], Lael M Yonker[2#], Galit Alter[1#]

Supplemental Materials

Fig. S1 to S5

Table S1 and S2

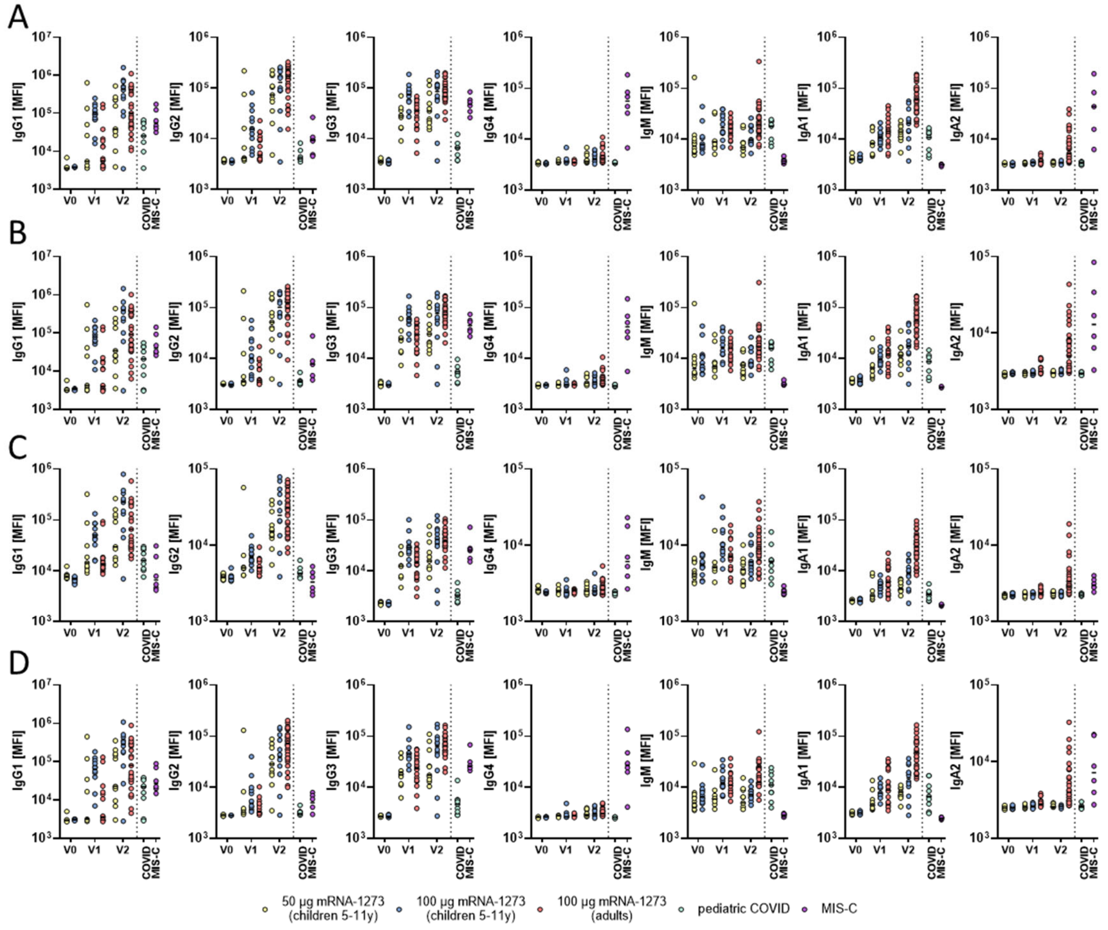

Figure S1. Vaccine induced antibody responses to different severe acute respiratory syndrome coronavirus 2 (SARS-CoV-2) variants of concern (VOC). Titers of IgG subclass (IgG1, IgG2, IgG3, IgG4) and additional isotypes (IgM, IgA1, IgA2) specific to the receptor binding domain (RBD) of (**A**) wild-type, (**B**) alpha, (**C**) beta, and (**D**) delta VOCs are shown. Samples were collected from children before ($V0_{50\ \mu g}$: n=12; $V0_{100\ \mu g}$: n=12), after the first ($V1_{50\ \mu g}$: n=9; $V1_{100\ \mu g}$: n=12) or after the second ($V1_{50\ \mu g}$: n=9; $V2_{100\ \mu g}$: n=11) dose of vaccine or in adults receiving two 100 µg doses of vaccine (V2: n=14). Samples were also collected from convalescent pediatric COVID-19 (n=9) or MIS-C (n=6) patients. COVID-19, coronavirus disease 2019; MIS-C, multisystem inflammatory syndrome in children; MFI, median fluorescence intensity. Horizontal bars indicate median.

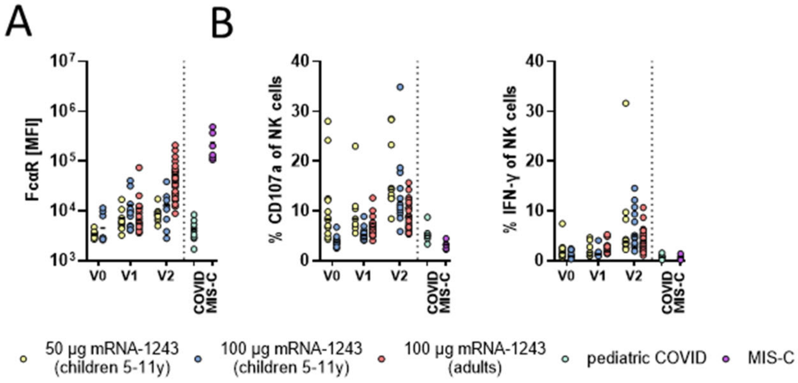

Fig. S2. Univariate comparisons across vaccine profiles in children and adults. (**A**) The plot shows the binding of wild-type SARS-CoV-2-specific IgA antibodies to Fc α receptor (FcαR) by Luminex. Horizontal bars indicate mean. (**B**) The plots show the antibody-dependent natural killer (NK) cell activating (ADNKA) responses in children and adults to the SARS-CoV-2 wild-type spike protein; ADNKA was measured by CD107a and interferon (IFN)-γ expression. Sample sizes are the same as shown in fig. S1.

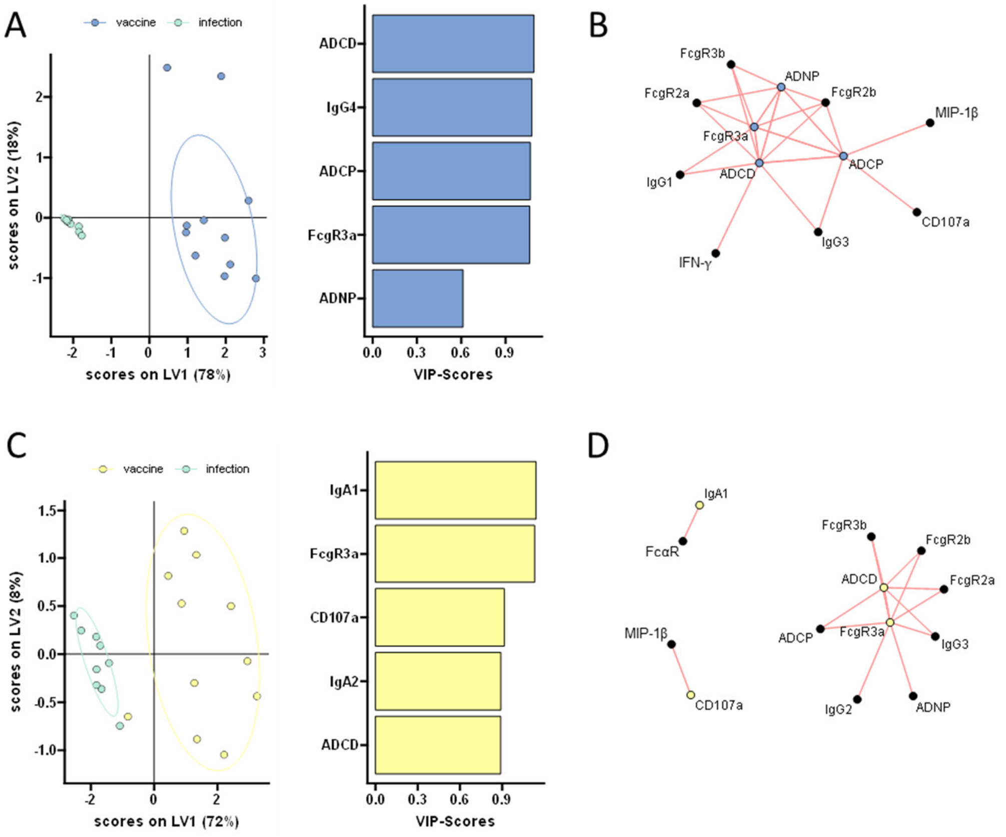

Fig. S3. Distinct humoral profiles were observed in infected and vaccinated children. A machine learning model was built to compare SARS-CoV-2 profiles in infected children and children receiving (**A and B**) 100 μg or (**C and D**) 50 μg vaccine doses (vaccine$_{50\ \mu g}$: n=11; vaccine$_{100\ \mu g}$: n=12; convalescent pediatric COVID (infection): n=9). (**A and C**) A minimal set of least absolute shrinkage and selection operator (LASSO)-selected SARS-CoV-2 spike protein-specific features (left panels) were first selected and used to discriminate pediatric vaccine responses (at V2) from infection (acute COVID) in children. Only five features were sufficient to completely separate the two groups (right panels). (**B and D**) A co-correlate network was used to define additional features that differed between children with COVID-19 and children who

received 100 μg (B) or 50 μg (D) doses of vaccine. ADCD, antibody-dependent complement deposition; ADNP, antibody-dependent neutrophil phagocytosis; ADCP, antibody-dependent monocyte phagocytosis; MIP-1β, macrophage inflammatory protein 1 beta; IFN-γ, interferon gamma.

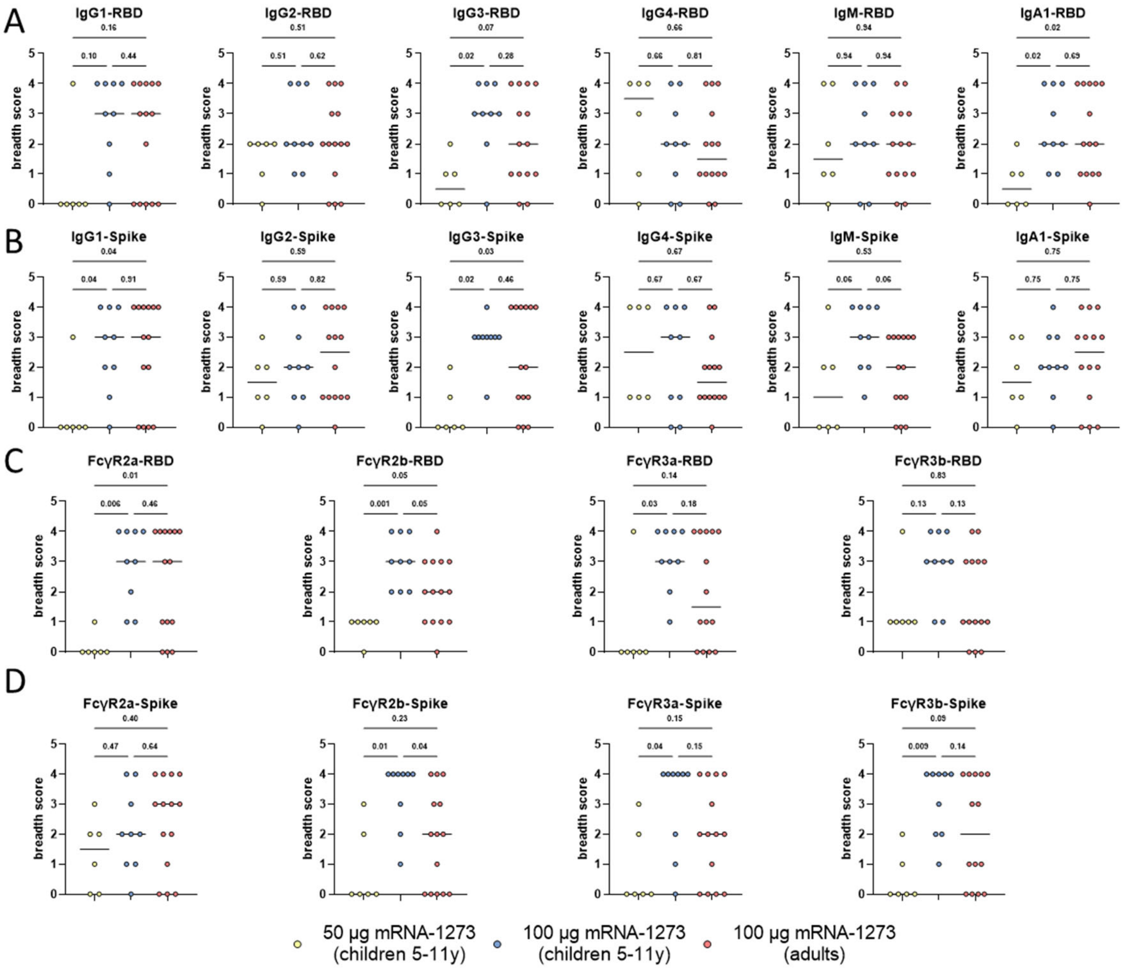

Fig. S4. VOC breadth score. VOC breadth scores were calculated for individual antibody features ($n_{50\ \mu g}$=6, $n_{100\ \mu g}$=9) or adults (n=14) at V2. (**A and B**) subclass and isotype binding and (**C and D**) FcγR binding for RBD (A and C) and spike protein (B and D). Horizontal bars indicate mean. P-values calculated by a non-parametric Kruskal-Wallis test after Benjamini-Hochberg correction for multiple correction are shown.

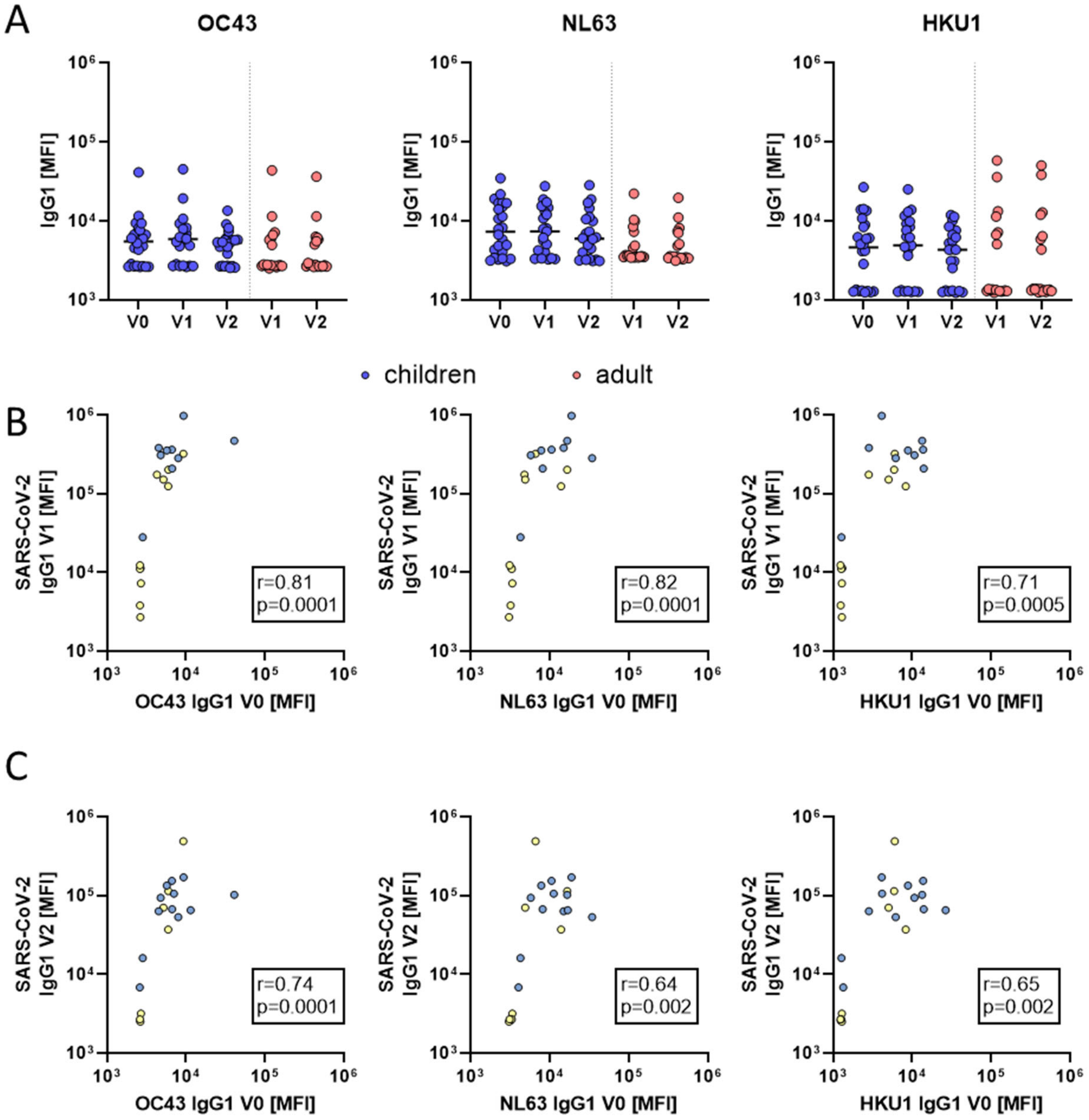

Fig. S5. IgG1 binding titers to other circulating human coronaviruses. (**A**) IgG1 subclass binding titers to human coronavirus OC43, HKU1, or NL63 spike protein were analyzed by Luminex in children (n_{V0} = 24, n_{V1} = 21, n_{V2} = 23) and adults (n = 19) during the observation period. **(B and C**) Spearman correlations are shown for pre-existing (V0) OC43, NL63, or HKU1 IgG1 titers and SARS-CoV-2 D614G spike protein responses at V1 (B) or V2 (C). Spearman correlation coefficient (r) and p-value are shown in the boxes.

Table S1. Pediatric participant demographics and clinical information (NA: not available)

	**mRNA-1273 children (50 µg)**	**mRNA-1273 children (100 µg)**	**mRNA-1273 adults (100 µg)**	**pediatric COVID**	**MIS-C**
Age, years, median (max, min)	*8 (6, 11)*	*9 (7, 11)*	33 (20, 55)	*5 (1,9)*	*10 (3,22)*
Sex, male, n (%)	*6 (50)*	*7 (58)*	13 (39)	*4 (44)*	*5 (83)*
Race n (%)					
White<br>Black<br>Asian<br>other	10 (83)<br>0<br>2 (17)<br>0	7 (58)<br>1 (8)<br>2 (17)<br>2 (17)	**NA**	3 (33)<br>2 (22)<br>0<br>4 (44)	2 (33)<br>1 (17)<br>0<br>3 (50)
Ethnicity n (%)					
Hispanic<br>Non-Hispanic<br>Unknown	3 (25)<br>9 (75)<br>0	3 (25)<br>8 (67)<br>1 (8)	**NA**	3 (33)<br>5 (56)<br>1 (11)	4 (67)<br>1 (17)<br>1 (17)
known SARS-CoV-2 pre-exposure, n positive (%)	*0*	*0*	0	*9 (100)*	*6 (100)*
Sample timepoints, n					
V0<br>V1<br>V2	12<br>9<br>11	12<br>12<br>12	-<br>19<br>33	9[1]	6[2]

Table S2. Post-vaccine symptom assessment survey. Ten children in the 50 µg cohort and 12 children in the 100 µg cohort completed the survey at each time point.

		mRNA-1273 children (50 µg)		mRNA-1273 children (100 µg)	
**Symptom Report**	**Side Effect**	**Following Dose #1 (V1) (n=10)**	**Following Dose #2 (V2) (n=10)**	**Following Dose #1 (V1) (n=12)**	**Following Dose #2 (V2) (n=12)**
**Local Reaction**	Pain/swelling at injection site	7 (70%)	2 (20%)	8 (66.7%)	5 (41.7%)
Constitutional	Fever	2 (20%)	7 (70%)	6 (50%)	6 (50%)
	Fatigue/excessive tiredness	2 (20%)	4 (40%)	4 (33.3%)	5 (41.7%)
	Chills	0 (0%)	3 (30%)	1 (8.3%)	0 (0%)
Neurological	Headache	1 (10 %)	4 (40 %)	4 (33.3%)	3 (25.0%)
Musculoskeletal	Muscle/body aches	0 (0%)	2 (20%)	2 (16.7%)	2 (16.7%)
Dermatologic	Rash	0 (0%)	1 (10%)	1 (8.3%)	2 (16.7%)
Gastrointestinal	Nasua/Vomiting	0 (0%)	1 (10%)	1 (8.3%)	1 (8.3%)
	Abdominal Pain	0 (0%)	0 (0%)	0 (0%)	0 (0%)
	Diarrhea	0 (0%)	0 (0%)	0 (0%)	0 (0%)
Cardiovascular	Persistent chest pain or pressure	0 (0%)	0 (0%)	0 (0%)	0 (0%)
	Heart palpitations	0 (0%)	0 (0%)	0 (0%)	0 (0%)
Respiratory	Sore throat	0 (0%)	0 (0%)	0 (0%)	0 (0%)
Mucocutaneous	Eye redness	0 (0%)	0 (0%)	0 (0%)	0 (0%)
Other	Includes anxiety/upset, nose-bleeds	0 (0%)	0 (0%)	1 (8.3%)	2 (16.7%)
No side effects	Patient reported no side effects	1 (10 %)	2 (20%)	1 (8.3%)	3 (25.0%)
